# Extraction of enhanced, ultrashort laser pulses from a passive 10-MHz stack-and-dump cavity

**DOI:** 10.1007/s00340-016-6574-x

**Published:** 2016-12-02

**Authors:** Sven Breitkopf, Stefano Wunderlich, Tino Eidam, Evgeny Shestaev, Simon Holzberger, Thomas Gottschall, Henning Carstens, Andreas Tünnermann, Ioachim Pupeza, Jens Limpert

**Affiliations:** 1 Institute of Applied Physics, Abbe Center of Photonics, Friedrich-Schiller-Universität Jena, Albert-Einstein-Str. 15, 07745 Jena, Germany; 2 Active Fiber Systems GmbH, Wildenbruchstr. 15, 07745 Jena, Germany; 3 Helmholtz-Institute Jena, Fröbelstieg 3, 07743 Jena, Germany; 4 Max-Planck-Institute of Quantum Optics, Hans-Kopfermann-Str. 1, 85748 Garching, Germany; 5 Department of Physics, Ludwig-Maximilians-Universität München, Am Coulombwall 1, 85748 Garching, Germany; 6 Fraunhofer Institute for Applied Optics and Precision Engineering, Albert-Einstein-Str. 7, 07745 Jena, Germany; 7 Present Address: Menlo Systems GmbH, Am Klopferspitz 19a, 82152 Martinsried, Germany

**Keywords:** Pulse Energy, Input Pulse, Diffraction Efficiency, Switching Rate, Nonlinear Phase

## Abstract

Periodic dumping of ultrashort laser pulses from a passive multi-MHz repetition-rate enhancement cavity is a promising route towards multi-kHz repetition-rate pulses with Joule-level energies at an unparalleled average power. Here, we demonstrate this so-called stack-and-dump scheme with a 30-m-long cavity. Using an acousto-optic modulator, we extract pulses of 0.16 mJ at 30-kHz repetition rate, corresponding to 65 stacked input pulses, representing an improvement in three orders of magnitude over previously extracted pulse energies. The ten times longer cavity affords three essential benefits over former approaches. First, the time between subsequent pulses is increased to 100 ns, relaxing the requirements on the switch. Second, it allows for the stacking of strongly stretched pulses (here from 800 fs to 1.5 ns), thus mitigating nonlinear effects in the cavity optics. Third, the choice of a long cavity offers increased design flexibility with regard to thermal robustness, which will be crucial for future power scaling. The herein presented results constitute a necessary step towards stack-and-dump systems providing access to unprecedented laser parameter regimes.

## Introduction

A number of visionary applications like laser wake-field acceleration of elementary particles [1] or space debris removal [2] ask for a dramatically improved performance of femtosecond laser systems with high repetition rates [3]. In particular, Joule-level pulse energies at average powers in the multi-kilowatt regime with diffraction-limited beam quality are required. This combination of parameters greatly exceeds the capabilities of today’s laser systems, and the scalability of the average and of the pulse peak power of single-aperture amplifier solutions does not suffice these demands [4–7]. Current limitations which need to be overcome are mainly caused by thermal or nonlinear effects in the amplifier media [8, 9]. Recently, multi-aperture spatial combining approaches have emerged as one possibility to circumvent these limitations [10, 11]. Additionally, temporal combining techniques aimed at artificially extending the stretched pulse duration and, thus, overcoming pulse peak power limitations have been successfully demonstrated. Among those, the most straightforward approach is the so-called divided-pulse amplification (DPA) [12]. Here, in order to reduce the peak power-related limitations, each pulse is split into several temporally separated replicas before the final amplification stage and recombined afterwards. Alternatively, the creation of temporal replicas can be avoided, if a pulse train with a much higher repetition rate is amplified and subsequently temporally combined to achieve the repetition rate demanded by the application. Here, the general idea is to increase the pulse peak power at the cost of a reduced repetition rate by temporally stacking successive pulses after their amplification. One implementation of this approach, which we refer to as stack and dump (SND), is to superpose amplified pulses in an enhancement cavity (EC) and periodically extract them using a fast and efficient switch [13, 14].

Passive ECs have been subject to intensive research and development for several decades [15–17]. They are employed for a multitude of intracavity optical conversion processes such as high-harmonic generation [18, 19] or inverse Compton scattering [20]. Due to the energy enhancement in such a cavity, average powers in the MW range [21] and multi-GW peak power levels [22] are achievable within the cavity at multi-MHz repetition rates. In 2002 and 2003, the extraction of pulses from such an enhancement cavity was proposed [23] and demonstrated at around 80-MHz with nJ-level, picosecond pulses by the Ye and Hänsch groups [24, 25]. In 2004, slightly stretched femtosecond pulses were first enhanced and then extracted from a 100-MHz cavity [26]. Recently, concepts making use of the vast potential of ECs as stacking devices for stretched ultrashort pulses were published [13, 27].

In this paper, we demonstrate the SND scheme in a 30-m-long EC, corresponding to a length increase of a factor of 10 over the state of the art. Towards tapping the full potential of ECs as stacking devices for ultrashort pulses, this constitutes a crucial design criterion relaxing the thermal stress in the switch and in the cavity optics [28] and allowing for longer times between successive pulses. The EC supported a steady-state power enhancement factor exceeding 200 and was seeded with a 10-MHz repetition-rate train of 3-µJ pulses. The cavity enabled the enhancement of strongly stretched pulses (~1.5 ns). A systematic investigation of different dumping rates was performed with an intracavity acousto-optic modulator (AOM). Pulses with the accumulated energy of up to 65 input pulses, i.e. 0.2 mJ, were extracted at 30 kHz. These pulses were recompressed to the initial duration of 800 fs, demonstrating the feasibility of SND with strongly stretched pulses and energies surpassing previous results by three orders of magnitude. These results, even if not stating new laser parameter records on their own, constitute the first milestone towards a power-scalable device and, thus, are a necessary step towards the first stack-and-dump system providing truly unprecedented laser parameters. Peak power-related and thermal limitations of this technique are discussed.

## Cavity set-up and steady-state enhancement

**Fig. 1:**
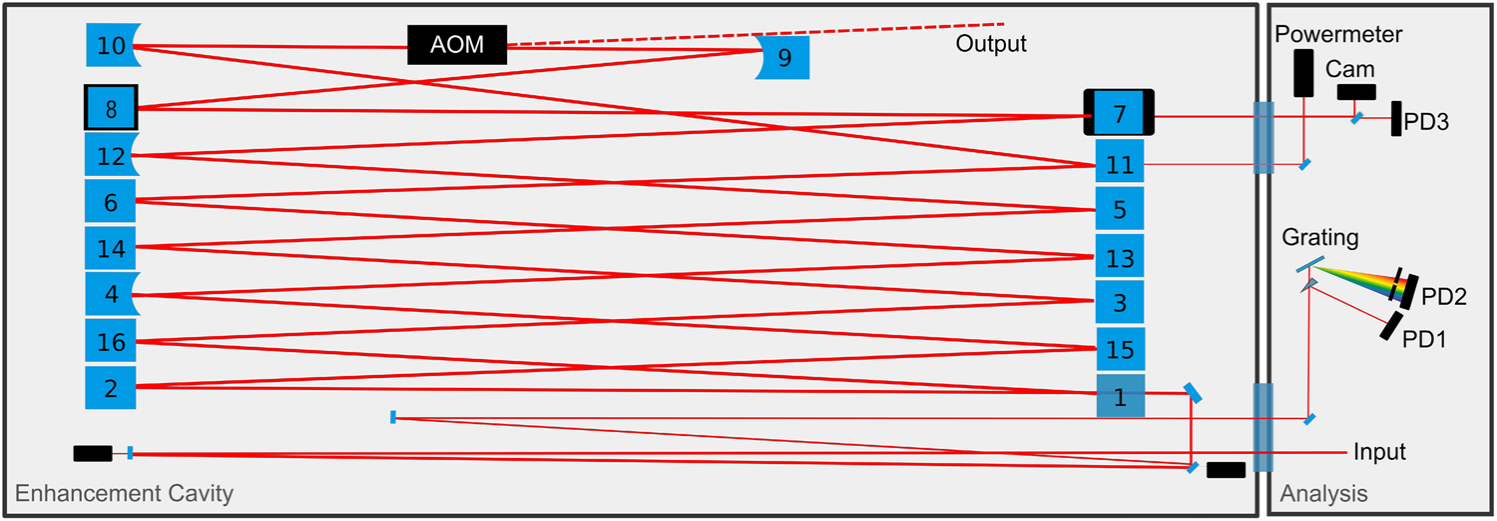
Schematic of the EC. The 30-m-long EC consists of one input-coupling mirror (1, *R* = 99%) and 15 highly reflective (HR) mirrors (2–16). Two of the HR mirrors are curved (4, 12) in order to form a stable resonator (see Fig. 2a). The mirrors 8 and 9, which are plane for the steady-state experiment, are replaced by curved ones once the AOM is inserted for the non-steady-state experiment (see Fig. 2a). The beams transmitted through mirrors 7 and 11 are sent to diagnostics such as a camera (Cam) and photodiodes (PD). The photodiode behind the grating is used for the Pound–Drever–Hall stabilization scheme [29]

In a first experiment, the steady-state behaviour of the EC without the AOM was investigated. The 30-m-long ring cavity (Fig. 1) was seeded with an average power of *P*_in_ = 30 W at 10-MHz repetition rate. Hence, the energy of the incoming pulses was *E*_in_ = 3 µJ. The pulses, spectrally centred around *λ* = 1038 nm were stretched to ~1.5-ns duration (measured at the −5-dB level of the maximum pulse intensity). A telescope was used to match the spatial mode of the incoming beam to the TEM_00_ mode of the EC, providing a measured overlap of *U* = 80% (which includes the spatial and the spectral overlap).

This set-up allowed for an energy enhancement factor

1 $$ V = E_{\text{circ}} /E_{\text{in}} , $$

of 213, where *E*_circ_ is the energy of the pulse circulating in the EC. During steady-state operation, the intracavity average power was measured to be 6.4 kW, corresponding to an energy of the circulating pulses of 0.64 mJ. The round-trip losses *L* within the cavity were estimated to be 0.22%.

**Fig. 2:**
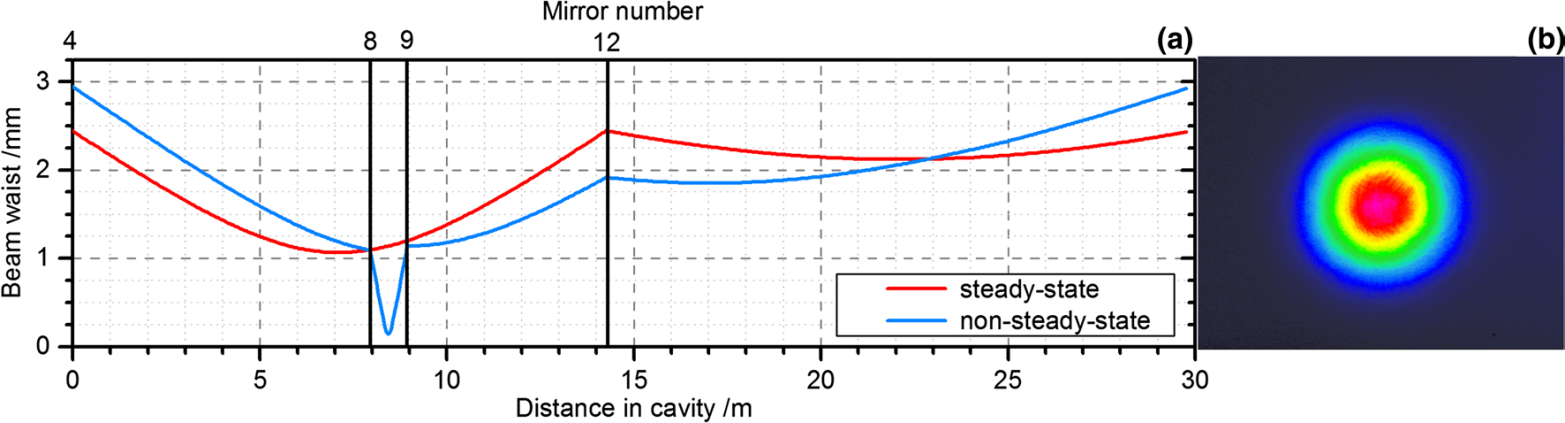
**a** Cavity caustic for the steady-state experiment (*red*) and the non-steady-state experiment (*blue*). The positions of the curved mirrors 4, 12 and 8, 9 (only for the non-steady-state experiment) are indicated by *vertical lines*. **b** Transmitted beam profile of the intracavity beam at an enhancement factor of 213

The power enhancement was mainly limited by the reflectivity of the input-coupling (IC) mirror *R* = 99%, which did not fulfil the impedance matching condition (*R* = 1 − *L*) corresponding to optimum steady-state enhancement. However, this IC was purposely chosen to allow for a comparison to the non-steady-state experiment, in which a higher reflectivity would have been disadvantageous due to the additional losses induced by the switching device. The calculated caustic and the measured steady-state beam profile are shown in Fig. 2.

## Non-steady-state operation: pulse extraction

For the dumping of pulses from the EC, an AOM was employed because of its simple implementation and fast switching times. The commercially available AOM (MQ80-A0.7-L1030.1064 from AA Opto-Electronic) used here offered an active aperture with a diameter of 0.7 mm and had a thickness of 23.5 mm. The rise time of the acoustic waves in this AOM is 110 ns/mm and depends on the diameter of the beam *d*. Using a TTL trigger signal with the experimentally optimized duration of ~50 ns, single-pulse extraction was enabled. The facets were antireflection coated for wavelengths between 1030 and 1060 nm. The AOM introduced additional transmission losses of about 0.6% and hence increased the overall round-trip losses *L* to 0.9% and the achieved steady-state enhancement to about 90. The cavity caustic was modified with respect to the steady-state experiment (see Fig. 2) in order to achieve an appropriate spot size in the AOM. The plane cavity mirrors 8 and 9 were replaced by concave mirrors with *R*_8,9_ = 1000 mm to obtain a focus with a 1/e^2^-diameter of *d* = 0.3 mm within the AOM, leading to negligible clipping losses while still providing a diffraction efficiency *η*_diff_ of around 72%. Hence, the circulating pulse could only be partially extracted and the energy enhancement of the extracted pulses compared to the input pulses (short: extracted enhancement) can be defined as

2 $$ V_{\text{extr}} = \eta_{\text{diff}} \times V. $$

**Fig. 3:**
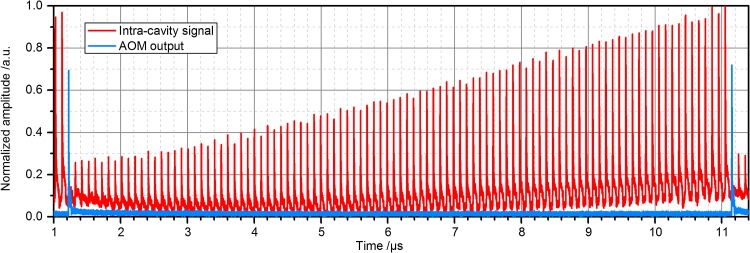
Exemplary measurement of the entire pulse build-up within the EC using an AOM with 72% diffraction efficiency and a switching rate of 100 kHz (100 stacked pulses)

The energy that remains in the cavity after the extraction (see Fig. 3) changes the subsequent build-up cycle. After adjusting the mode-matching telescope, the beam overlap *U* was similar to the steady-state case. Dumping via the AOM was triggered synchronously to the laser repetition frequency after an integer number of pulses, employing a gate function just wide enough for a single pulse. The intracavity and output signals are shown exemplarily in Fig. 3 for 100 stacked pulses.

The switching rate (the repetition rate of the extracted pulses) can be calculated as

3 $$ f_{\text{switch}} = f_{\text{rep}} /N, $$

were *N* represents the number of stacked pulses. As mentioned before, 28% of the enhanced pulse energy remained inside the cavity at the end of each build-up cycle, affecting the subsequent build-up. However, after a certain number of round trips an equilibrium is reached and the extracted enhancement after a large number of build-up cycles can therefore be described analytically with the following equation (derived similarly to [30]):

4 $$ V_{\text{extr}} = \eta_{\text{diff}} \frac{{(1 - R)\left( {\sqrt {AR}^{N} - 1} \right)^{2} }}{{\left( {\sqrt {AR} - 1} \right)^{2} \left[ {1 - \sqrt {AR}^{N} \sqrt {1 - \eta_{\text{diff}} } } \right]^{2} }}, $$

where *A* = 1 − *L* is the round-trip attenuation. This equation was used for the simulations of the cavity behaviour. The power at the output port of the AOM was measured for different switching rates and used to determine the cavity efficiency

5 $$ \eta = \eta_{\text{stack}} \times \eta_{\text{diff}} = P_{\text{out}} /P_{\text{in}} , $$

which describes how much of the input average power is conserved during the increase in the pulse energy of a laser system via stack and dump. The energy of the extracted pulses can be derived as

6 $$ E_{\text{out}} = P_{\text{out}} /f_{\text{switch}} . $$

The extracted enhancement can therefore also be written as

7 $$ V_{\text{extr}} = \eta_{\text{diff}} \times V = E_{\text{out}} /E_{\text{in}} . $$

**Fig. 4:**
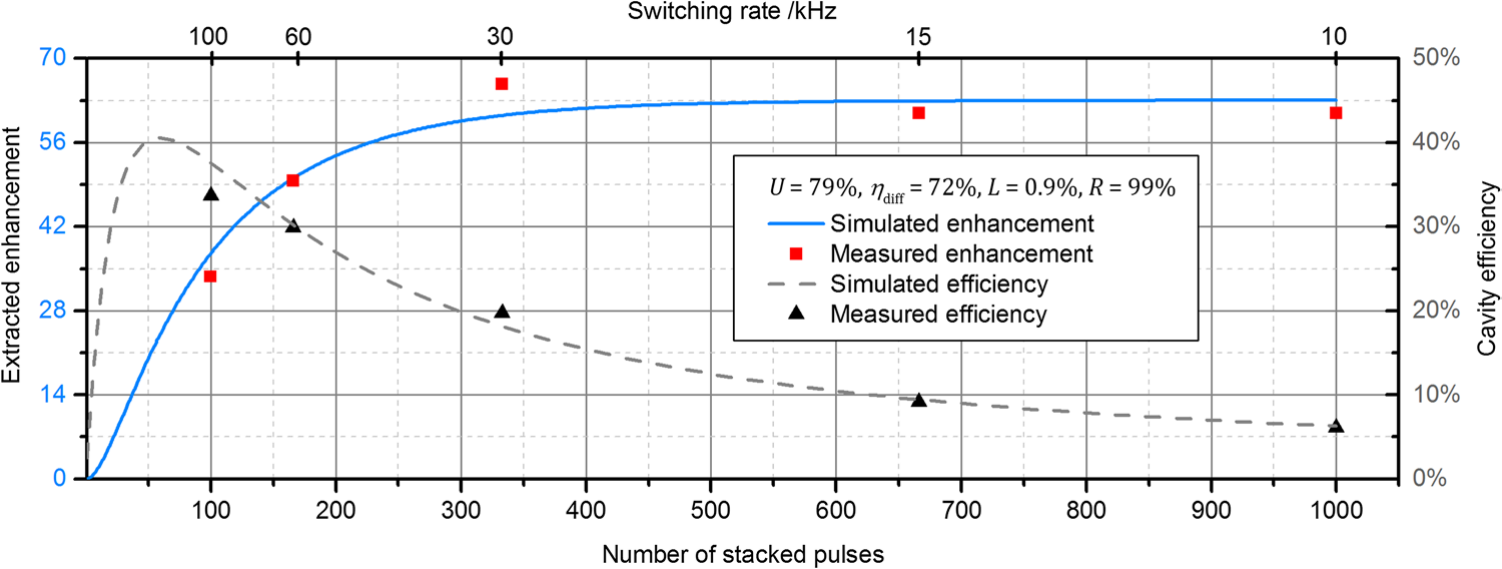
Extracted enhancement and cavity efficiency for various switching rates along with the theoretical predictions calculated from Eq. 4

Figure 4 shows a plot of the measured extracted enhancement and cavity efficiency over the number of stacked pulses and the switching rate, compared to theoretical predictions. In agreement with the theory, a smaller number of stacked pulses lead to a smaller extracted enhancement at a given input coupler reflectivity. The measured efficiency shows a clear maximum for 100 stacked pulses, reaching 34%. When the number of stacked pulses is increased, a saturation of the enhancement sets in and the efficiency therefore drops continuously.

The efficiency as well as the extracted enhancement can be further optimized by adapting the input coupler reflectivity R for each switching rate as discussed in [13]. For a given input coupler reflectivity, the optimum working point in terms of the switching rate depends on whether the highest pulse energy or the highest efficiency is desired. The small deviations of the measured values from the ones predicted by theory (Fig. 4) are caused by variations in the alignment, slightly changing the overlap between the incoming beam and the cavity mode. It is noteworthy that the stabilization of the oscillator to the cavity was barely affected by the dumping process. Only at the highest investigated switching rates, the partial dumping occasionally leads to a collapse of the lock.

**Fig. 5:**
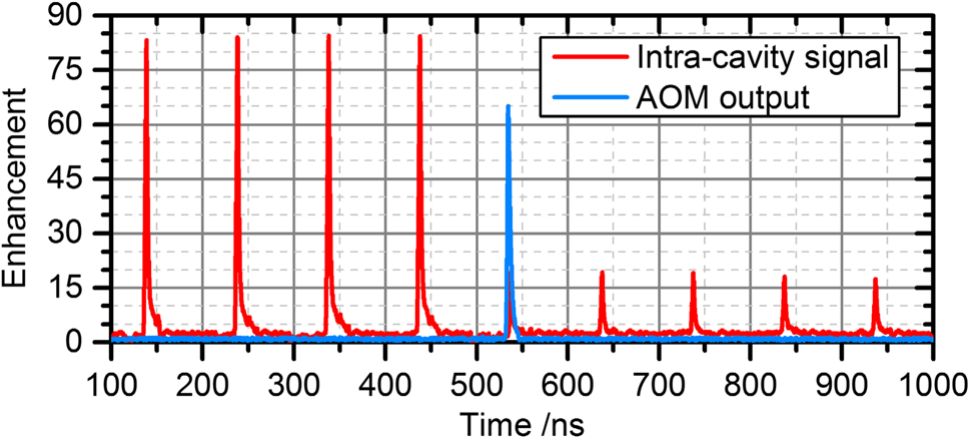
Photodiode signals of the intracavity pulse (*red*) and of the output pulse (*blue*) for a switching rate of 30 kHz, revealing the extraction of a single pulse. A fraction of the pulse remains in the cavity due to the limited diffraction efficiency of the AOM

A switching rate of 30 kHz offered the highest extracted enhancement (~65), corresponding to an output pulse energy of 197 µJ. Figure 5 shows the photodiode signal of the intracavity pulses and the output of the system during this measurement. The dumped 30-kHz pulses were recompressed using a grating compressor with an efficiency of ~80% resulting in an energy of 0.16 mJ.

**Fig. 6:**
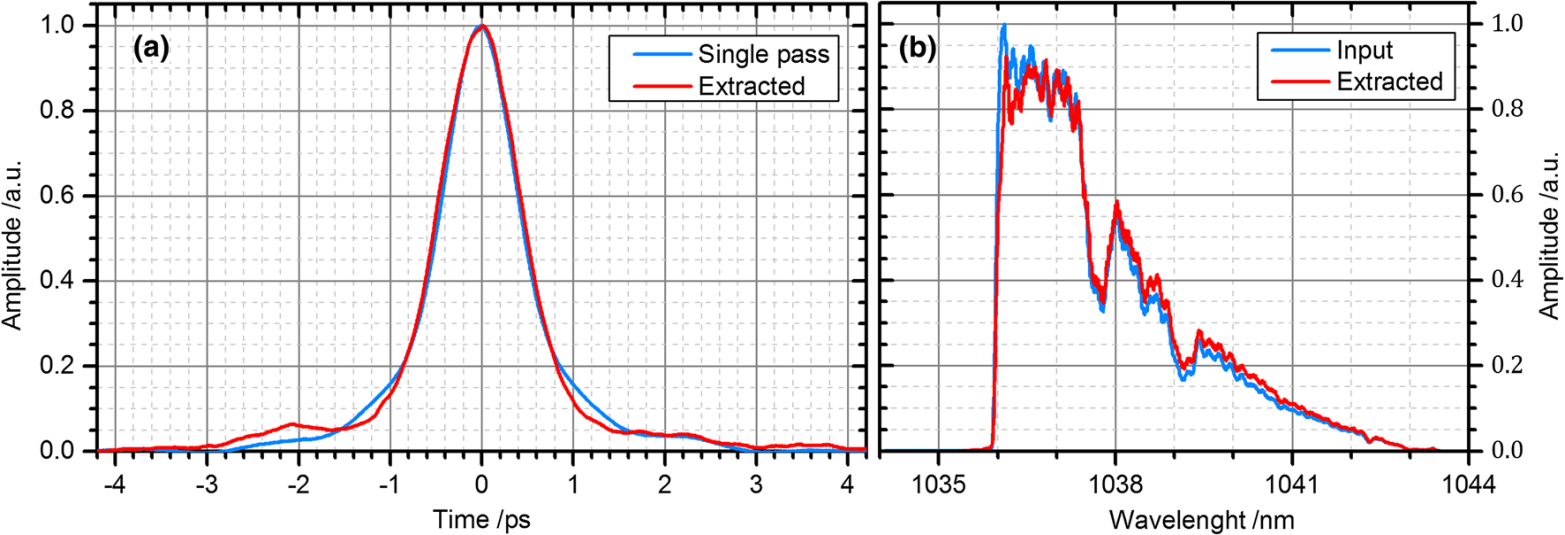
**a** Autocorrelation traces (AC) of a diffracted pulse in the single-pass set-up (zeroth order of the AOM blocked) and of an extracted pulse during cavity operation. In the latter case, the shape is slightly different and the AC duration increased from 1.00 to 1.08 ps. The duration of the extracted pulse was estimated to be around 800 fs. **b** Spectrum of the signal before the EC (*blue*) and of the extracted pulse (*red*), both clearly showing the hard-cut of the stretcher at 1036 nm

Figure 6 depicts a measurement of the autocorrelation and of the spectrum of the pulses. Additionally, a reference autocorrelation (Fig. 6a) was acquired in a single cavity-pass set-up without any input-coupling mirror and hence without any enhancement. Using the measured output spectrum and the spectral phase, the duration of the enhanced pulses was estimated to be around 800 fs. This is only slightly longer than the input pulses, which is also confirmed by the almost identical spectrum of the input and of the extracted pulses (see Fig. 6b).

**Fig. 7:**
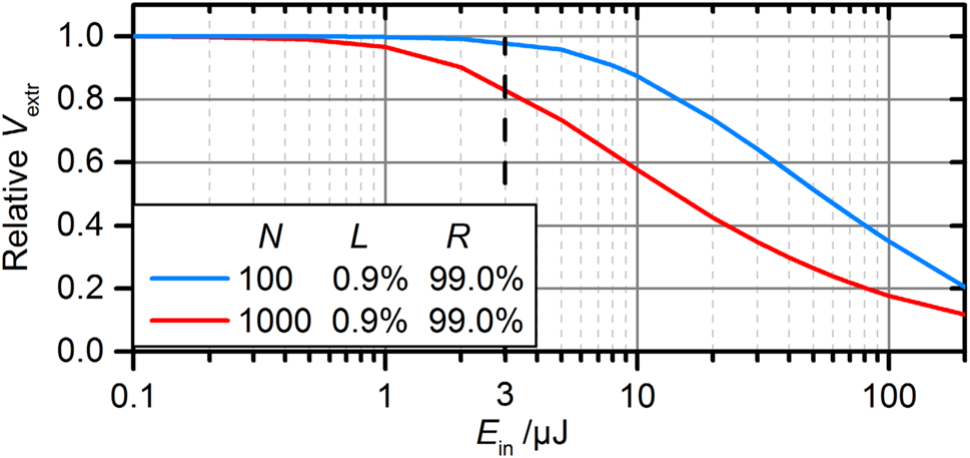
Relative decrease in the extracted enhancement due to the nonlinear phase acquired via SPM in the AOM. Simulated for the pulse build-up in a 10-MHz cavity for the smallest and largest number of stacked pulses as a function of the input pulse energy (diameter in AOM of 0.4 mm, stretched pulse durations of 1.5 ns)

In order to estimate the limitations induced by the nonlinear phase associated with self-phase-modulation (SPM) in the AOM, the pulse build-up in the cavity was numerically simulated step by step under consideration of this effect. At a certain threshold, the reduced overlap of the electric field of the intracavity pulse with the seeding pulse will lead to a drop in the stacked pulse energy and therefore in the extracted energy. The simulation was carried out for different switching rates, using the input spectrum (Fig. 6b), a length of the AOM of 23.5 mm, a spot diameter in the AOM of 0.4 mm (as required in a 10-MHz cavity), a stretched pulse duration of 1.5 ns and a diffraction efficiency of the AOM of 72%. As shown in Fig. 7, for the experimental setting presented here (*E*_in_ = 3 µJ, black dotted line), the occurrence of SPM in the AOM was already starting to limit the extracted pulse energy. For 3 µJ of input energy and between 100 and 1000 stacked pulses, the possible extracted enhancement was slightly reduced compared to the SPM-free case. Due to the larger number of round trips and hence a greater acquired nonlinear phase, the effect becomes more critical when *N* is increased.

The spot size in the AOM is crucial for the peak intensity, but on the other hand it is determined by the required switching time for a given cavity length. To achieve an efficient enhancement at an increased input energy, using an AOM as a switching device, it is hence necessary to extend the cavity length. This measure offers the additional benefit that for a certain desired switching rate (i.e. output repetition rate) the required number of stacked pulses is lower for longer cavities (see Eq. 3) which decreases the magnitude of the acquired nonlinear phase due to lower average round trips of the pulses. Naturally a sweet spot has to be found in the trade-off between a high enhancement and a minimized nonlinear phase.

## Conclusion and outlook

The experiments presented here constitute a first demonstration that pulse stacking of stretched fs pulses in a 10-MHz cavity is a promising route towards increasing the pulse peak power of high-average-power ultrafast laser systems. An extracted enhancement of 65, delivering pulse energies of 0.16 mJ at 30 kHz with a pulse duration of 800 fs, demonstrates a significant improvement over previous results [24–27]. To achieve this progress, it was necessary to lengthen the cavity in order to allow for a longer switching time with the additional benefit of a cavity mode that is less sensitive to thermal effects [28]. Other temporal pulse combining techniques, like divided-pulse amplification [31, 32], are limited in the number of combined pulses due to an increasing complexity. Stack and dump currently constitutes the most promising way for the superposition of a large number of pulses.

In the next experimental step, the AR-coated AOM will be exchanged for a Brewster-cut AOM and a state-of-the-art seed system [33] will be used to deliver up to 1 mJ at 2-MHz repetition rate with a spectrum enabling pulse durations below 300 fs. Our simulations under consideration of SPM-related effects in the AOM show that at the corresponding cavity length of 150 m an extracted enhancement of around 50 should be feasible by stacking 100 pulses. Together with a good management of the thermal lenses [34] that may occur in the cavity, these modifications will enable the extraction of 50-mJ pulses at 20-kHz repetition rate with a cavity efficiency of 50%, conserving 1 kW average power. Finally, a purely reflective switch [13, 14] overcoming AOM-related limitations is highly desirable to further increase the performance and efficiency of the system to Joule-class pulse energy and multi-kW average powers.
